# Physicochemical and Morphological Changes in Long-Grain Brown Rice Milling: A Study Using Image Visualization Technologies

**DOI:** 10.3390/foods13193033

**Published:** 2024-09-24

**Authors:** Xiwu Jia, Rong Dong, Xuan Chen, Zhan Wang, Hongjian Zhang, Wangyang Shen

**Affiliations:** 1 Department of Food Science and Engineering, Wuhan Polytechnic University, Wuhan 430023, China; jiaxiwu212@126.com (X.J.); 17786182428@163.com (R.D.); chenxuan_1986.163@163.com (X.C.); wangzh_whpu@163.com (Z.W.); 2 Key Laboratory for Deep Processing of Major Grain and Oil, Ministry of Education, Wuhan Polytechnic University, Wuhan 430023, China; 3 Hainan Institute of Grain and Oil Science, Qionghai 571400, China; 21110832000013@hainanu.edu.cn

**Keywords:** degree of milling, appearance quality, micro-CT, volatile compounds

## Abstract

This study evaluated the changes in physicochemical properties and appearance quality of long-grain rice during the grinding process using image technologies and aimed to provide reference for future research. The brown rice milling process was divided into three stages, and flatbed scanning, scanning electron microscopy (SEM), X-ray micro-computed tomography (micro-CT), low-field nuclear magic resonance (LF-NMR), and headspace–gas chromatography–ion mobility spectrometry (HS–GC–IMS) were employed to examine the physicochemical and volatile properties of the samples. Results revealed a continuous increase in the degree of milling, with a broken rice rate and a whiteness value increasing by 50.84% and 21.13%, respectively, compared with those during the initial stage; dietary fiber and vitamin B1 contents were reduced by 54.41% and 66.67%, respectively. The image results visualized showed that the cortex of brown rice was gradually peeled off with the increase in milling degree; the cortical thickness was gradually reduced, the endosperm was gradually exposed, and the surface was smoother and shinier. T_2_ populations exhibited a shift toward longer relaxation times, followed by a decrease in relaxation time during the milling process. Additionally, 31 target compounds impacting rice flavor, mainly ketones, alcohols, and esters, were identified, and the concentration of volatile substances in the B region decreased with the reduction in the bran layer; the concentration of volatile substances in the C region provided rice flavor, which increased with the milling process. This study showed changes in the physicochemical properties and appearance quality of long-grain brown rice during milling. Furthermore, the use of various image processing techniques offers significant insights for optimizing processing parameters and enhancing overall quality and taste.

## 1. Introduction

Rice is one of the most essential staple foods globally consumed, with a pleasing aroma and flavor as well as nutritional value [1,2]. Among its varieties, long-grain indica rice attracts attention for its distinctive characteristics. The grains are typically slender, oval, or oblong and frequently exhibit a more transparent color, with some varieties showing a light-yellow color; the length of long-grain indica rice usually exceeds 6.0 mm, with some varieties reaching over 7.5 mm [3]. Long-grain indica rice is favored for its attractive appearance, strong water absorption, significant expansion upon cooking, high yield, and clear nonsticky grains; it is ideal for producing high-grade rice, sushi, and rice noodles, making it popular among consumers and highly valued in the market [4]. However, the bran layers, which are good sources of nutrients and phytochemicals, would be gradually removed during the milling process, resulting in nutrient loss [5]; additionally, long-grain indica rice is longer, the viscosity is smaller, the rice quality is brittler than the japonica rice, and it is easy to be broken during the milling process, thereby resulting in broken rice and affecting the processing efficiency and output [6,7]. Therefore, the control of long-grain indica rice processing precision is important for rice nutritional quality, palatability, heavy metal content, and processing loss.

Although studies including those by Zhao et al. [8] have explored the effects of rice variety and processing accuracy on nutritional, sensory, and taste qualities using principal component analysis to reveal the varied and occasionally limited effects of grinding, and Ren et al. [9] have used scanning electron microscopy (SEM) to analyze the appearance and morphology of japonica rice at different milling stages, dividing the process into four distinct phases in a laboratory experiment, a gap in understanding the specific milling dynamics of long-grain indica rice at the rice production line still exists. In addition, Liu et al. [10] proposed a product quality classifier based on sparse multi-core least-squares support vector machine to solve the low confidence classification problem of uneven data distribution; this method can be applied to the production line of food processing enterprises to automatically classify (or identify) the production quality of rice. Roy et al. [11] investigated the influence of processing conditions on the energy consumption and quality of short- and long-grain rice; the results showed that the lower grinding degree resulted in more food nutrients (lipids and dietary fiber (DF)) being retained in rice. For long-grain indica rice, a special rice variety, few studies have been conducted to explore its rice milling process, which is limited to the influence of the rice milling degree on nutritional chemical composition, cooking quality, and processing characteristics. On the other hand, most of the studies lack comprehensive data integration, which affects the accuracy of process improvement; and the existing research technology may not be able to adapt to the needs of different environments and large-scale production, which requires researchers to integrate all kinds of data and conduct in-depth analysis to optimize the processing technology and develop adaptive technologies that can adapt to different production conditions and scales. In the field of rice processing, the in-depth study of image detection technology is of great significance for intelligent online monitoring of the quality of materials in the process of rice production. Image analysis technology is used to study the export materials in the rice-milling process comprehensively and systematically, which can provide valuable reference materials for automatic and intelligent rice production [12].

Therefore, image visualization technologies were used to investigate the rice milling process in this study. The objectives of this study were as follows: (1) to clearly characterize the changes in morphology, quality, and physicochemical properties of long-grain indica rice during the large-scale milling process, thereby providing scientific basis and technical support for the rice processing technology and product quality; and (2) to provide rice processing companies with new perspectives aimed at optimizing their production processes and technologies, improving the automation and intelligence level of rice processing traditional production line, while developing more efficient grinding techniques for improving product quality and yield.

## 2. Materials and Methods

### 2.1. Materials

Long-grain indica rice (Ewan 10) samples were obtained from an industrial production line in Hubei KongHong Grain and Oil Food Co., Ltd. (Huanggang, China), which adopted three rice milling technology. After husking, the brown rice go through three horizontal emery-roller rice whitener (MNSW 30 F × 2, Hunan Chenzhou Grain & Oil Machinery Co., Ltd. (Chenzhou, China)) marked stage1, stage2, stage3. The capacity of horizontal emery-roller rice whitener was 8–12.5 t/h, and the processing flow was controlled at 8 tons per hour in this study. One kilogram of brown rice was collected from the rice huller export, and 1 kg of other samples was obtained from three different milling stages at the production line, respectively. The samples were sealed and placed in a desiccator until the experiments were conducted.

### 2.2. Degree of Milling (DOM)

Brown rice was milled through three processes on the production line; the weight of each process before and after milling was recorded, and the milling reduction rate was calculated using the following equation:

$$DOM=\frac{M_{0}-M}{M_{0}}\times 100\%$$

where DOM is the milling reduction rate, %; M_0_ is the mass of brown rice before milling; and M is the mass of rice after milling [13,14].

### 2.3. Ratio of Broken Kernels, Rice Color, and Appearance Image Acquisition

The ratio (%) of broken kernels, rice color, and appearance image acquisition in milled rice was measured by manual analysis using a rice appearance detection system (CanoScan 9000 F MarkII, Wuhan, China).

### 2.4. DF and Vitamin B1 Contents

Soluble DF (SDF) and insoluble DF (IDF) contents were analyzed using a Megazyme assay kit (Megazyme, Bray, Ireland). An approximately 1 g brown rice powder sample was collected, added to the buffer, and digested with α-amylase and protease. The dry weight of the sample was used for determining the IDF content. The filtrate was mixed with the washing solution and precipitated with hot ethanol. The sample was filtered through the Gooch crucible, and the residue was dried to determine the SDF content as a percentage of dry weight. Total DF was calculated by adding IDF and SDF [15].

The content of water-soluble vitamin B1 in samples was analyzed using overpressure liquid chromatography. Water-soluble vitamins were extracted from rice flour by acid and enzyme hydrolysis and subsequently centrifuged after cooling. The supernatant was collected into an HPLC automatic sampler vial. The water extract was injected into a reversed-phase C18 HPLC column. The fluorescence of riboflavin was determined, and thiamine was converted to chromium sulfide by an alkaline potassium ferricyanide column [16]. The vitamin B1 content was measured. The vitamin standard was purchased from Sigma Aldrich (St. Louis, MO, USA) (thiamine hydrochloride, vitamin B1).

### 2.5. Microstructure Analysis

S-3000 SEM (HITACHI, Tokyo, Japan) was used for microscopic scanning with a 15.00 kV voltage. The rice sample was placed flat on the sample stage, observed under an electron microscope at a 1000× *g* magnification, and scanned to analyze its appearance.

### 2.6. X-ray Micro-CT Analysis

The NanoVoxel Scan system of Tianjin Sanying Precision Instrument Co., Ltd. (Tianjin, China) was used for observing the seed-coat thickness distribution of rice grains at different grinding stages. First, the test sample was put into the sample chamber for scanning, and the sample was rotated to obtain the projection map. To obtain the reconstructed section image from the projection data, various algorithms were used; subsequently, the CT section map was reconstructed for data processing to obtain the final image [17]. The scanning mode, tube voltage, and tube current were microchamber high-resolution mode, 60 KV, and 0.2 mA, respectively; the transverse and axial fields of view were 5 and 2 mm, respectively; the reconstruction type was FDK; and the pixel size was 0.005 × 0.005 × 0.005 mm^3^.

### 2.7. Water Distribution and Mobility in the Samples

A low-field (21 MHz) 1 H nuclear magic resonance spectrometer (model: NMI20-040V-I; Niumag Electronics Technology, Shanghai, China) operating at 32.0 ± 0.1 °C was used for examining the range of proton molecular mobility by measuring the transverse (T2) relaxation time and the fraction of the signal amplitude of protons in each part of T2. The method by Liu et al. [18] was slightly modified by taking 10 g of rice samples obtained at different milling stages, wrapping them in plastic wrap to prevent water loss during the NMR experiment, and placing them in a 40 mm NMR tube for follow-up testing. T2 (transverse relaxation times) curves were obtained using a Carr–Purcell–Meiboom–Gill (CPMG) pulse sequence with a 4 s recycle delay, eight scans, and a 0.1 ms interpulse spacing.

### 2.8. Volatile Analysis

The volatile profile of samples was determined by an ion mobility spectrometry system (IMS) (model: FlavourSpec^®^ 1H1-00053; G.A.S, Dortmund, Germany) equipped with an Agilent 6890 N gas chromatograph (model: 6890 N; Agilent Technologies, Palo Alto, CA, USA) using a nonpolar SE-54-CB GC column. For better reproducibility, the device was equipped with an automatic sampler unit (CTC Analytics AG, Zwingen, Switzerland) that can be directly sampled from the headspace using a 1 mL air-tight heated syringe. Tritium 3 H, which provided a 6.5 keV radiation energy, was the ionization source of the IMS. The carrier gas and drift gas were N2 (99.999%) with a 101 kPa drift pressure (ambient pressure).

Each sample (3.0 g) was transferred into a 20 mL vial and incubated for 15 min at 60 °C. A 500 µL headspace volume was automatically injected into the injector (80 °C, splitless mode) using a heated syringe (80 °C). The carrier gas passed through an HS–GC–IMS injector transferring the sample into the GC column at a programmed flow as follows: 2, 15, 80, 130, and 150 mL/min for 2, 8, 10, 5, and 5 min, respectively. Subsequently, the analytes were pushed into an ionization chamber for ionization before being driven into the drift region via a shutter grid and finally passed into an IMS detector. The flow rate of the drift gas was set at 250 mL/min. The retention index (RI) of each compound was calculated using n-ketones C4–C9 as external references. Volatile compounds were identified by comparing the RI with the drift time (the time it took for ions to reach the collector through a drift tube, in milliseconds) of the standard in the GC–IMS library [19].

### 2.9. Statistical Analysis

All experiments were conducted in approximately 3–5 parallel experiments. Statistical Package for the Social Sciences (version 25, IBM, Armonk, NY, USA) was used for part of the data analysis. Data were expressed as means ± standard deviations. To detect treatment effects, one-way analysis of variance was applied. The significance level tested was at least *p* ≤ 0.05. Graph analyses were performed using Origin 9.0 software.

## 3. Results and Discussion

### 3.1. DOM, Broken Rice, Color, Content of DF, and Vitamin B1 Changes in Rice during the Milling Process

The variations in DOM, broken rice rate, whiteness, DF content, and vitamin B1 content as the degree of rice milling intensifies are presented in Table 1. The milling process was divided into three stages for analysis. Progressively, as the milling process advances, the DOM continuously increased from 1.26% to 8.35%; concurrently, the broken rice rate and the whiteness value increased by 50.84% and 21.13%, respectively, compared with that during the initial rice milling stage; conversely, DF and vitamin B1 contents were reduced by 54.41% and 66.67%, respectively. During the milling process, the bran layer of the brown rice was progressively peeled off, accounting for the observed increase in the DOM. In the rice milling process, rice grains not only bear the shear force and milling pressure from the rice mill but also collide with other rice grains or the internal structure of the rice mill during continuous tumbling in the rice mill to be subjected to the action of forces in all directions; these forces will continuously destroy the internal structure of the rice grain, reduce its structural strength, and make it breakable, thereby resulting in the increased crushing rate of rice [20]. As the milling progresses and the brown rice’s bran layer decreases, the underlying white translucent endosperm becomes increasingly visible, thereby leading to the enhancement in the rice’s whiteness. The bran layer of brown rice is rich in DF and vitamins, particularly vitamin B complex [21]. The gradual removal of this layer during the milling process consequently results in reduced DF and vitamin B1 contents.

### 3.2. Rice Appearance Image Analysis

**Figure 1:**
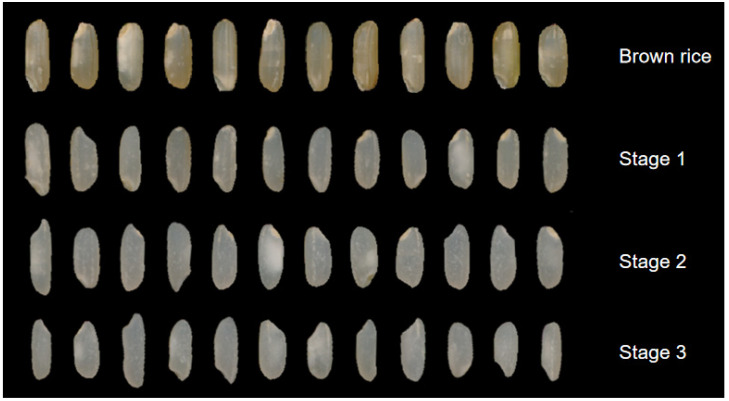
The morphology of rice grains at different milling stages.

The appearance characteristics of rice grains at various stages of the milling process are shown in Figure 1. The initial brown rice, post hulling, exhibited a brown color, and the surface of the grains was smooth, with a waxy luster, and featured distinct longitudinal grooves, since the rice surface has five longitudinal furrows; furthermore, the depth of the furrow varies with the rice variety [5]. The embryos were completely intact and discernible on the surface, with some grains presenting a pronounced white belly, which was attributed to the presence of a loose silky endosperm structure and a white opaque cross-section caused by the gaps between the starch granules and the cell wall. Following the first milling stage, the whiteness of the rice grains significantly increased, a very small number of embryos were peeled off, the longitudinal groove on the surface of the rice grains became less obvious, and a very small number of rice grains had white belly. In the second milling stage, most of the embryos were peeled off, and the whiteness increased compared with that during the first stage. Only a few rice grains had abdominal white phenomenon; however, it was not obvious. In the third and final milling stages, the embryos of the rice grains were all markedly peeled off, the whiteness increased, no white belly was noted, and the surface was smooth and shiny. The deep yellow color of the brown rice was mainly caused by the rich carotenoid and flavonoid contents in the cortex. These compounds are common natural pigments in plants, which not only provide brown rice a brown color but also have antioxidant effects that are beneficial to health [22]. However, after milling, the cortical thickness of the brown rice gradually decreased, and the internal white transparent endosperm became gradually exposed, thereby increasing the whiteness of the rice.

### 3.3. Microstructure Properties

**Figure 2:**
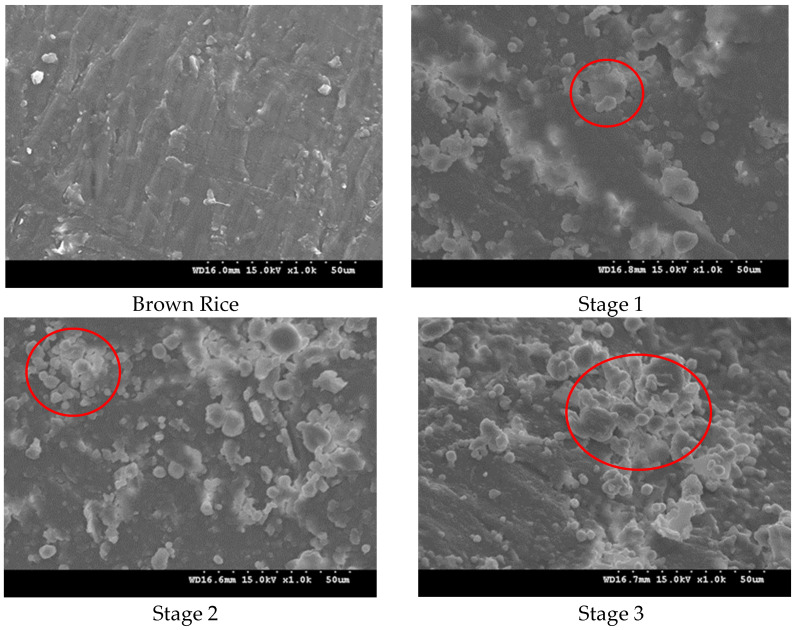
Scanning electron microscope image of the rice surface (The particles in the red circles represent rice starch granules and broken rice bran).

A SEM analysis of the rice grains at various stages of the milling process is shown in Figure 2. The sample of brown rice has a uniform ground-glass-style surface, while samples at different process stages have uneven seed-coat residual protrusions, exposed aleurone layers, or concave endosperm structures; a scattered broken epidermis on the surface with cracks in parts was also observed. This finding was because the cortex of brown rice contained a large number of fibers, and the small particles may be the rice bran particles and rice starch granules produced by the force on the surface of brown rice during the hulling process. During the first rice milling stage, no filamentous fiber was observed, and the pores were reduced; however, the surface remained uneven, and some areas may be smoother than other areas. This finding was because the bran layer in some areas was completely removed when the rice bran was peeled off. Obvious cracks were noted on the surface of the rice grain, and granular materials with uneven size and shape were also observed, which may be caused by the decrease in the surface structural strength of the rice grain due to the force during the milling process, thereby resulting in cracks. The particles with uneven size and shape were endosperm particles or exfoliated aleurone layer particles that were gradually exposed after the brown rice cortex fell off [23]. During the second rice milling stage, the number of small endosperm particles increased, and the surface of the rice grains was smoother than that during the first stage. This finding was because with the increase in the rice milling degree, the endosperm became gradually exposed and was broken by force, thereby decreasing in size, and most of the bran layer was removed, thereby increasing the surface flatness. During the third rice milling stage, the number of endosperm particles increased, and the surface of the rice grains became increasingly smooth. This finding was because of the completely removed bran layer, completely exposed endosperm, and strong endosperm structure [24]. Variations in the surface appearance of the rice, such as bumps, scratches, scattered starch, and roughness, clearly show mechanical damage caused by the rice milling machine during the milling process [9].

### 3.4. Micro-CT Analysis

**Figure 3:**
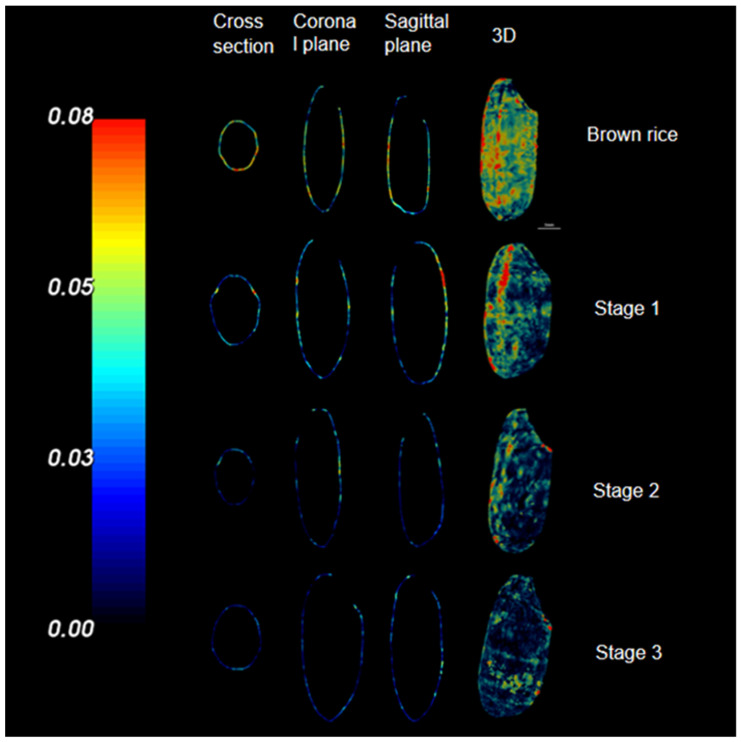
Microscopic computed tomography images of the rice-seed-coat thickness distribution during milling.

X-ray micro-CT is a nondestructive method for examining internal structures based on the differences in the strength of X-ray attenuation within a scanned material [25]. This method reconstructs three-dimensional (3D) images based on a series of two-dimensional radiographs and can perform micrometer-scale visualization. The application of micro-CT in this study has elucidated the distribution of rice-seed-coat thickness at various stages of the milling process, as illustrated in Figure 3. The visual representation is such that the color intensity in micro-CT images corresponds to the seed-coat thickness, with brighter areas indicating thicker regions and darker areas signifying thinner regions. CT images revealed the changes in the seed-coat thickness distribution during rice milling from four aspects, namely cross-section, coronal plane, sagittal plane, and 3D imaging. Notably, the seed coat of brown rice was the thickest, particularly in the abdominal groove, which was more substantial than those in other areas. During the first rice milling stage, the micro-CT images revealed a uniform seed-coat peeling, except for a minor portion in the groin area. The second milling stage was characterized by the selective removal of a few layers in the abdominal furrow, whereas the third stage involved further grinding to peel off the majority of the remaining skin, intentionally avoiding complete removal to prevent excessive processing that can result in nutrient loss [26].

**Figure 4:**
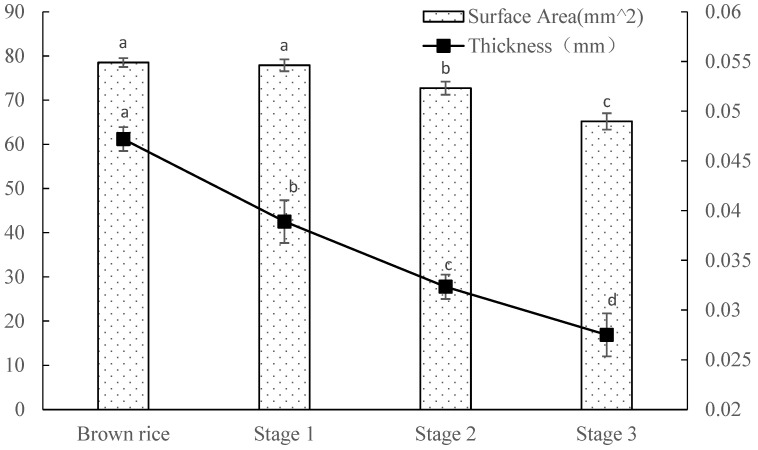
Surface area and skin thickness of rice during milling. Small letters indicate significant differences among samples at different process stage (*p* < 0.05).

The changes in the rice grain surface area and skin thickness throughout the milling stages are depicted in Figure 4. Interestingly, in the first stage, the surface area of the rice grains remained largely unchanged, whereas the thickness of the skin layer was reduced by approximately 0.01 mm. This finding can be attributed to the rapid removal of the embryo, which developed a concave surface that does not significantly affect the overall surface area. In the subsequent stages, both the surface area and the thickness of the grain cortex exhibited a decreasing trend, as the cortex progressively thinned postembryonic removal, culminating in a reduced surface area [20]. Ultimately, the cortical thickness stabilized at approximately 0.0275 mm, which was consistent with the results in CT images.

### 3.5. Molecular Mobility (^1^H NMR) Analysis

The T_2_ relaxation time distribution was obtained by the multiexponential fitting of the CPMG relaxation curve continuously distributed during brown rice milling. The 1H T_2_ quasi-continuous distributions of the brown rice milling process are presented in Table 2. Three T_2_ populations were observed in all samples and were subsequently named, starting from the shortest to the longest relaxation time, 1, 2, and 3, respectively. Populations 1, 2, and 3 represented protons relaxing at 0.1–1 ms (T_21_), 1–100 ms (T_22_), and >100 ms (T_23_), respectively. Population 1 was considered to be water, which was tightly bound to proteins; population 2 was water that was bound to starch and soluble substances; and population 3 was water that was exchanged between starch and protein [27].

T_21_, T_22_, and T_23_ represent the transverse relaxation time of strongly bound, weakly bound, and free water in the samples, respectively. The peak area (T_21_, T_22_, and T_23_) was calculated by integrating the relaxation signals, namely A_21_, A_22_, and A_23_, which represent the relative proportion of strongly bound, weakly bound, and free water in the samples, respectively [28]. As an emerging technology for nondestructive testing, LF-NMR can reflect the motion characteristics of water molecules in a system by detecting various parameters, including proton relaxation behavior and amplitude. The shorter the relaxation time, the more stable the water state, and the tighter the binding time between the water and the substrate; the longer the relaxation time, the better the water mobility and the higher the degree of freedom observed [29].

The relaxation time of T_21_ remained unchanged, and no right shift was noted. The relaxation time of T_22_ decreased after increasing to the second rice milling stage, whereas that of T_23_ decreased following the first rice milling stage. This finding demonstrated that T_22_ and T_23_ first bonded and became less compact in the process and subsequently became tighter again. The content of A_21_ in bound water was much greater than those in A_22_ and A_23_, and with the increase in the rice milling degree, the proportion of strongly bound water A_21_ increased by 6.02%, 4.31%, and 0.59%; the proportion of weakly bound water A_22_ initially increased by 311.18% and subsequently decreased by 44.11% and 34.05%; and the proportion of free water A_23_ decreased by 87.06%, 23.68%, and 57.76%. These results indicated that the water in brown rice was mainly bound water and primarily distributed in the endosperm of rice grains, the immobile water was mainly distributed in the aleurone layer of brown rice, and the free water was mainly distributed in the peel and seed coat of brown rice. The changes in water-binding strength may be related to the structural strength of brown rice itself, and the binding force between the skin and endosperm of brown rice is smaller than that of the endosperm; therefore, when the peel and seed coat are rolled down in the first stage and the aleurone layer is removed in the second stage, changes in structural strength will be noted [30]. Additionally, the moisture-binding strength may be influenced by the temperature during rice milling. The increase in rice temperature during the rice milling process may lead to the evaporation of water on the surface of the rice grains, making the surface of the rice grains dry, while the internal water may be retained. This temperature change may lead to the uneven distribution of water inside and outside the rice grains, thereby affecting the moisture-binding strength [31].

### 3.6. Volatile Component Analysis

The volatile components of rice during the milling process were analyzed using HS–GC–IMS. The chromatographic visualization, presented against a blue backdrop, indicates the identity of each detected volatile organic compound beneath the corresponding column. The color intensity within these columns is correlated with the substance concentration, wherein white signifies low levels, whereas yellow to deep red denotes increasing concentration gradients [32]. This spectral overview encapsulates the headspace constituents of the rice samples, providing a comprehensive profile of volatile emissions.

The results of the analysis identified 31 distinct volatile compounds, with aldehydes, alcohols, and esters being the most prevalent. These compounds exhibit a wide variation in type and concentration, playing a crucial role in shaping the flavor profile of brown rice throughout the milling process. Esters impart sweet, floral, and fruity notes, whereas aldehydes and alcohols contribute to a spectrum of green, grassy, fatty, fruity, and floral aromas [33,34]. For example, aldehydes are derived from lipid oxidation and contribute more to the aroma of cereal products owing to their low odor threshold (OAV) [19]. In the current study, volatile compounds originated from two primary sources: those inherent to the bran layer and those released through biochemical reactions, including fatty acid degradation and oxidation, protein breakdown, and the Maillard reaction.

**Figure 5:**
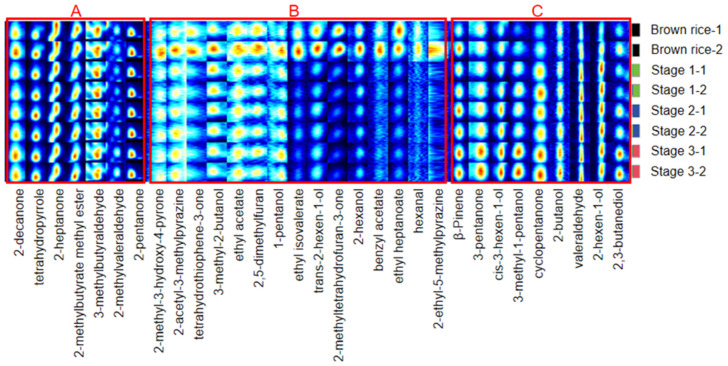
Volatile component changes in rice during the milling process. (Region A: volatile compounds and concentration did not obviously change; Region B: volatile compounds and concentration declined or disappeared, Region C: volatile compounds and concentration increased).

As observed in Figure 5, the volatile substances in region A of the spectrum do not change with the milling process of brown rice. In contrast, the volatile compounds in region B of the spectrum gradually declined or disappeared in concentration as milling progressed, potentially owing to the diminishing bran layer, which is associated with undesirable odors. Conversely, compounds in region C (β-Pinene, 3-pentanone, cis-3-hexen-1-ol, 3-methyl-1-pentanol, cyclopentanone, 2-butanol, valeraldehyde, 2-hexen-1-ol, and 2,3-butanediol) demonstrated an incremental increase, likely a consequence of the heightened milling temperature and duration, fostering lipid decomposition, oxidation, and chemical transformations within the bran layer, thereby generating volatiles that confer fruity and floral scents to the rice [35]. Notably, the concentration of specific compounds such as 2-methyl-3-hydroxy-4-pyrone and 2-acetyl-3-methylpyrazine decreased, whereas that of β-pinene and 3-pentanone increased, reflecting the complex interplay of biochemical changes during milling. Therefore, considering the influence of temperature on the release and transformation of volatile compounds, regulating the milling environment is imperative. To prevent excessive thermal degradation and ensure optimal flavor development, maintaining an appropriate temperature is essential, such as the air-cooling system, or cooling the water circulation system for rice milling temperature control is necessary [36].

## 4. Conclusions

With the progress in the rice milling process, the broken rice rate and whiteness value increased by 50.84% and 21.13%, respectively, compared with those during the initial stage; dietary fiber and vitamin B1 contents were reduced by 54.41% and 66.67%, respectively. Through appearance, micro-CT, and SEM, the changes in long-grain rice in the milling process were visualized; the cortex of brown rice was gradually peeled off with the increase in the milling degree; the cortical thickness was gradually reduced, the endosperm was gradually exposed, and the surface was smoother and shinier. T_2_ populations initially shifted toward longer relaxation times; subsequently, the relaxation time decreased during the milling. A total of 31 typical target compounds were identified using HS–GC–IMS, mainly including ketones, alcohols, and esters. Their changes during rice milling significantly affected the flavor of rice grains. The above-mentioned results showed that the physical and chemical properties and the appearance quality of long-grain brown rice significantly changed during milling, and the results of the analysis of different image techniques provided an important reference for the milling process of long-grain brown rice, which was helpful to optimize the processing parameters and obtain better quality and taste. To fully understand the grinding process of long-grain rice at rice large-scale production line and its impact on the quality of the final product, further research should explore the application of image visualization technologies at the rice large-scale production line to improving the automation and intelligence level of the rice processing industry.

## Figures and Tables

**Table 1:** Milling rate (%), broken rice rate (%), whiteness, dietary fiber content (g/100 g), and vitamin B1 content (mg/100 g) during milling.

	DOM (%)	Broken Rice Rate (%)	Whiteness	Dietary Fiber/(g/100 g)	VB1/(mg/100 g)
Brown rice	0	0.00	18.60 ± 0.17 ^a^	1.36 ± 0.11 ^a^	0.18 ± 0.01 ^a^
Stage 1	1.26 ± 0.05 ^a^	11.27 ± 1.12 ^a^	30.90 ± 0.36 ^b^	0.94 ± 0.04 ^b^	0.10 ± 0.03 ^b^
Stage 2	5.34 ± 0.34 ^b^	15.77 ± 1.36 ^b^	35.87 ± 0.75 ^c^	0.65 ± 0.01 ^c^	0.08 ± 0.02 ^b^
Stage 3	8.53 ± 1.02 ^c^	17.00 ± 1.05 ^c^	37.43 + 0.21 ^d^	0.62 ± 0.03 ^c^	0.06 ± 0.01 ^b^

Small letters indicate significant differences among samples at different process stage (*p* < 0.05).

**Table 2:** T_2_ relaxation time distribution and relative moisture content during milling.

	T_21_ (ms)	T_22_ (ms)	T_23_ (ms)	A_21_	A_22_	A_23_
Brown rice	0.66 ± 0.00	14.73 ± 4.01 ^a^	70.72 ± 4.93 ^a^	86.64 ± 0.05 ^a^	1.61 ± 0.20 ^a^	11.75 ± 0.15 ^a^
Stage 1	0.66 ± 0.00	61.51 ± 4.28 ^b^	328.25 ± 22.87 ^b^	91.86 ± 0.05 ^b^	6.62 ± 0.18 ^b^	1.52 ± 0.13 ^b^
Stage 2	0.66 ± 0.00	75.65 ± 0.00 ^c^	253.15 ± 52.23 ^c^	95.82 ± 0.14 ^c^	3.70 ± 0.11 ^c^	1.16 ± 0.22 ^c^
Stage 3	0.66 ± 0.00	37.89 ± 2.82 ^d^	232.53 ± 17.30 ^c^	96.39 ± 0.01 ^c^	2.44 ± 0.21 ^d^	0.49 ± 0.04 ^d^

Small letters indicate significant differences among samples with the same formulation at different storage times (*p* < 0.05).

## Data Availability

The original contributions presented in the study are included in the article, further inquiries can be directed to the corresponding author.

## References

[1] Sen S., Chakraborty R., Kalita P. (2020). Rice—Not just a staple food: A comprehensive review on its phytochemicals and therapeutic potential. Trends Food Sci. Technol..

[2] Summpunn P., Deh-Ae N., Panpipat W., Manurakchinakorn S., Bhoopong P., Donlao N., Rawdkuen S., Shetty K., Chaijan M. (2023). Nutritional Profiles of yoom noon rice from royal initiative of Southern Thailand: A comparison of white rice, brown rice, and germinated brown rice. Foods.

[3] Sandhu R.S., Singh N., Kaler R.S.S., Kaur A., Shevkani K. (2018). Effect of degree of milling on physicochemical, structural, pasting and cooking properties of short and long grain indica rice cultivars. Food Chem..

[4] Zhang Y., Li F., Huang K., Li S., Cao H., Xie J., Guan X. (2023). Structural changes of starch under different milling degrees affect the cooking and textural properties of rice. Food Chem. X.

[5] Longvah T., Boiroju N.K., Prasad V.S.S., Kumar K.O., Mangthya K., Sharma P., Ananthan R., Babu V.R. (2021). Nutrient diversity in 251 Indian rice germplasms and dietary nutrient supply through rice in rice based diets. LWT.

[6] Huang W.D., Miao X.X., Liu Z.M., Zhou K., Yang B.R., Li C.G. (2021). Effect of rice milling technology on milled rice yield of long-grain high quality indica rice. Hunan Agric. Sci..

[7] Kim S.Y., Lee H. (2012). Effects of quality characteristics on milled rice produced under different milling conditions. J. Korean Soc. Appl. Biol. Chem..

[8] Zhao S., Shi J., Cai S., Xiong T., Cai F., Li S., Chen X., Fan C., Mei X., Sui Y. (2023). Effects of milling degree on nutritional, sensory, gelatinization and taste quality of different rice varieties. LWT.

[9] Ren H., Qi S., Zhang L., Wang L., Huang J., Yang H., Ren C., Zhou W. (2021). Variations in the appearance quality of brown rice during the four stages of milling. J. Cereal Sci..

[10] Liu J., Tang Z., Zhang J., Chen Q., Xu P., Liu W. (2016). Visual perception-based statistical modeling of complex grain image for product quality monitoring and supervision on assembly production line. PLoS ONE.

[11] Roy P., Ijiri T., Okadome H., Nei D., Orikasa T., Nakamura N., Shiina T. (2008). Effect of processing conditions on overall energy consumption and quality of rice (*Oryza sativa* L.). J. Food Eng..

[12] Zareiforoush H., Minaei S., Alizadeh M.R., Banakar A. (2015). Potential applications of computer vision in quality inspection of rice: A review. Food Eng. Rev..

[13] Wang Z., Zhang M., Liu G., Deng Y., Zhang Y., Tang X., Li P., Wei Z. (2021). Effect of the degree of milling on the physicochemical properties, pasting properties and in vitro digestibility of simiao rice. Grain Oil Sci. Technol..

[14] Chen W., Li W., Wang Y. (2022). Evaluation of rice degree of milling based on Bayesian optimization and multi-scale residual model. Foods.

[15] Tuncel N.B., Yılmaz N., Kocabıyık H., Uygur A. (2014). The effect of infrared stabilized rice bran substitution on physicochemical and sensory properties of pan breads: Part I. J. Cereal Sci..

[16] Akhter K.T., Shozib H.B., Islam M.H., Sarwar S., Islam M.M., Akanda M.R., Siddiquee M.A., Mohiduzzaman M.D., Rahim A.T., Shaheen N. (2023). Variations in the major nutrient composition of dominant high-yield varieties (hyvs) in parboiled and polished rice of Bangladesh. Foods.

[17] Vicent V., Ndoye F.-T., Verboven P., Nicolaï B., Alvarez G. (2019). Effect of dynamic storage temperatures on the microstructure of frozen carrot imaged using x-ray micro-ct. J. Food Eng..

[18] Liu J., Liu Y., Wang A., Dai Z., Wang R., Sun H., Strappe P., Zhou Z. (2021). Characteristics of moisture migration and volatile compounds of rice stored under various storage conditions. J. Cereal Sci..

[19] Fan X., Jiao X., Liu J., Jia M., Blanchard C., Zhou Z. (2021). Characterizing the volatile compounds of different sorghum cultivars by both GC-MS and HS-GC-IMS. Food Res. Int..

[20] Fei J., Feng W., Jia F., Han Y., Chen P., Li A., Wang Y., Zhang J., Shen S., Hao X. (2023). The mechanism of bran layer removal in friction rice mills. Biosyst. Eng..

[21] Ping J., Ren L., Xiao H.T., Yan X.L., Du Q.Z. (2022). Effect of milling on proximate composition, γ-oryzanol, vitamin B1, polyphenolic, and bioaccessibility of phenolic of brown rice. J. Food Process. Preserv..

[22] Lamberts L., Delcour J.A. (2008). Carotenoids in raw and parboiled brown and milled rice. J. Agric. Food Chem..

[23] Lamberts L., Bie E.D., Vandeputte G.E., Veraverbeke W.S., Derycke V., Man W.D., Delcour J.A. (2007). Effect of milling on colour and nutritional properties of rice. Food Chem..

[24] Olatunde G.A., Atungulu G.G. (2018). Milling behavior and microstructure of rice dried using microwave set at 915 MHz frequency. J. Cereal Sci..

[25] Contardo I., Bouchon P. (2018). Enhancing micro-ct Methods to quantify oil content and porosity in starch-gluten matrices. J. Food Eng..

[26] Wang L., Duan W., Qian H., Zhang H., Qi X. (2016). Research status and development trend of brown rice food. Food Ferment. Ind..

[27] Pan Q., Zhou J., Shen W., Wang Z., Cai H., Jia X. (2022). Effect of extruded wheat bran on volatile and physicochemical properties of bread during its shelf life. J. Cereal Sci..

[28] Qiao C.-C., Tian X.-H., Wang L.-X., Jiang P., Zhai X.-T., Wu N.-N., Tan B. (2022). Quality characteristics, texture properties and moisture migration of fresh brown rice noodles under different storage and temperatures conditions. J. Cereal Sci..

[29] Cheng S., Zhang T., Yao L., Wang X., Song Y., Wang H., Wang H., Tan M. (2018). Use of low-field-nmr and MRI to characterize water mobility and distribution in pacific oyster (*Crassostrea gigas*) during drying process. Dry. Technol..

[30] Wood D.F., Siebenmorgen T.J., Williams T.G., Orts W.J., Glenn G.M. (2012). Use of microscopy to assess bran removal patterns in milled rice. J. Agric. Food Chem..

[31] Pan Z., Khir R., Thompson J.F. (2013). Effect of milling temperature and post milling cooling procedures on rice milling quality appraisals. Cereal Chem..

[32] Li Z., Sun X., Xu T., Dai W., Yan Q., Li P., Fang Y., Ding J. (2023). Insight into the dynamic variation and retention of major aroma volatile compounds during the milling of Suxiang Japonica rice. Food Chem..

[33] Din Y.L., Wang H.D., Tang G.W., Tian X. (2022). HS-GC-IMS and fingerprint of volatile organic compounds of chrysanthemum for 5 medicinal and dietary uses. Food Mach..

[34] de Melo Pereira G.V., de Carvalho Neto D.P., Júnior A.I., Vásquez Z.S., Medeiros A.B.P., Vandenberghe L. (2019). Exploring the impacts of postharvest processing on the aroma formation of coffee beans—A review. Food Chem..

[35] Gao C., Li Y., Pan Q., Fan M., Wang L., Qian H. (2021). Analysis of the key aroma volatile compounds in rice bran during storage and processing via HS-SPME GC/MS. J. Cereal Sci..

[36] Xian Q.H., Lin L., Zhen L.G., Zhi W.Z. (2020). Volatile compounds, affecting factors and evaluation methods for rice aroma: A review. Trends Food Sci. Technol..

